# Supplementary material for: Analysis of chi angle distributions in free amino acids via multiplet fitting of proton scalar couplings
Source: Magn Reson (Gott). 2024 Aug 19;5(2):103–20. doi: 10.5194/mr-5-103-2024 (PMC11570886; doi:10.5194/mr-5-103-2024)
Supplement: The supplement related to this article is available online at: https://doi.org/10.5194/mr-5-103-2024-supplement. [file mr-5-103-2024-supplement.zip › Syed2024/data/fit1d_fitnmr_output.tar.gz!/fit1d/gly/500/bmse000089_simulation_1_nmredata/1H/resonances_1d.pdf]

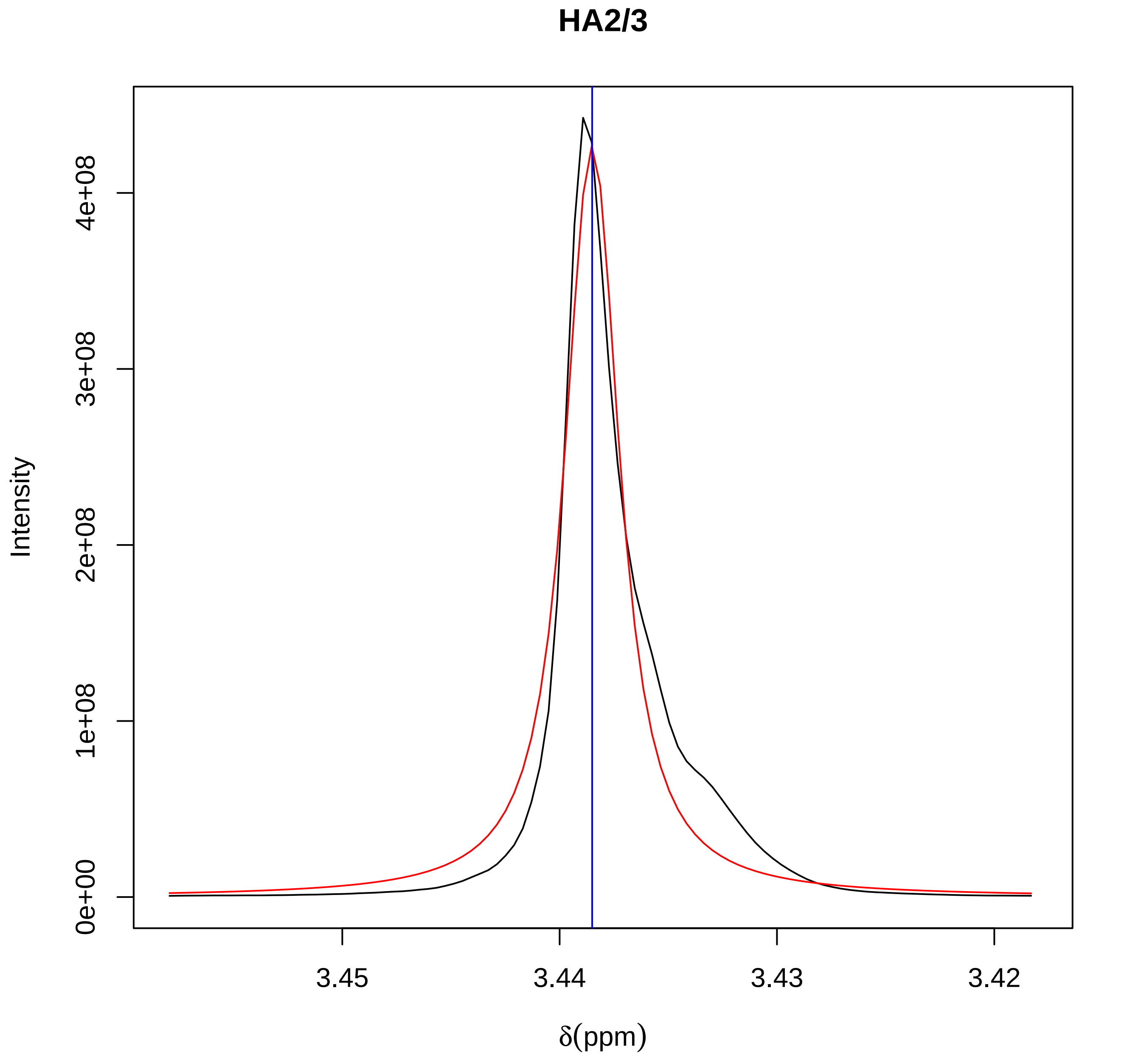
HA2/3
Intensity
0e+00
1e+08
2e+08
3e+08
4e+08
3.45
3.44
3.43
3.42
δ(ppm)
